# Microbial Contamination of Food: Probiotics and Postbiotics as Potential Biopreservatives

**DOI:** 10.3390/foods13162487

**Published:** 2024-08-08

**Authors:** Gordana Zavišić, Slavica Ristić, Saša Petričević, Drina Janković, Branka Petković

**Affiliations:** 1 Faculty of Pharmacy Novi Sad, University Business Academy in Novi Sad, Heroja Pinkija 4, 21101 Novi Sad, Serbia; 2 Faculty of Medicine, University of Belgrade, Pasterova 2, 11000 Belgrade, Serbia; slavicaristic8@gmail.com (S.R.); sasa_petricevic@yahoo.com (S.P.); 3 Vinča Institute of Nuclear Sciences—National Institute of the Republic of Serbia, University of Belgrade, Mike Petrovića Alasa 12-14, Vinča, 11351 Belgrade, Serbia; drinaj@vin.bg.ac.rs; 4 Institute for Biological Research “Siniša Stanković”—National Institute of the Republic of Serbia, University of Belgrade, Bulevar Despota Stefana 142, 11108 Belgrade, Serbia; janac@ibiss.bg.ac.rs

**Keywords:** food, microbial contamination, probiotics, postbiotics, preservatives, food safety

## Abstract

Microbial contamination of food and alimentary toxoinfection/intoxication in humans are commonly caused by bacteria such as *Salmonella* spp., *Escherichia coli*, *Yersinia* spp., *Campylobacter* spp., *Listeria monocytogenes*, and fungi (*Aspergillus*, *Fusarium*). The addition of probiotic cultures (bacterial strains *Lactobacillus* and *Bifidobacterium* and the yeast *Saccharomyces cerevisiae var. boulardii*) to food contributes primarily to food enrichment and obtaining a functional product, but also to food preservation. Reducing the number of viable pathogenic microorganisms and eliminating or neutralizing their toxins in food is achieved by probiotic-produced antimicrobial substances such as organic acids (lactic acid, acetic acid, propionic acid, phenylacetic acid, and phenyllactic acid), fatty acids (linoleic acid, butyric acid, caproic acid, and caprylic acid), aromatic compounds (diacetyl, acetaldehyde, reuterin), hydrogen peroxide, cyclic dipeptides, bacteriocins, and salivabactin. This review summarizes the basic facts on microbial contamination and preservation of food and the potential of different probiotic strains and their metabolites (postbiotics), including the mechanisms of their antimicrobial action against various foodborne pathogens. Literature data on this topic over the last three decades was searched in the *PubMed*, *Scopus*, and *Google Scholar* databases, systematically presented, and critically discussed, with particular attention to the advantages and disadvantages of using probiotics and postbiotics as food biopreservatives.

## 1. Introduction

**Figure 1:**
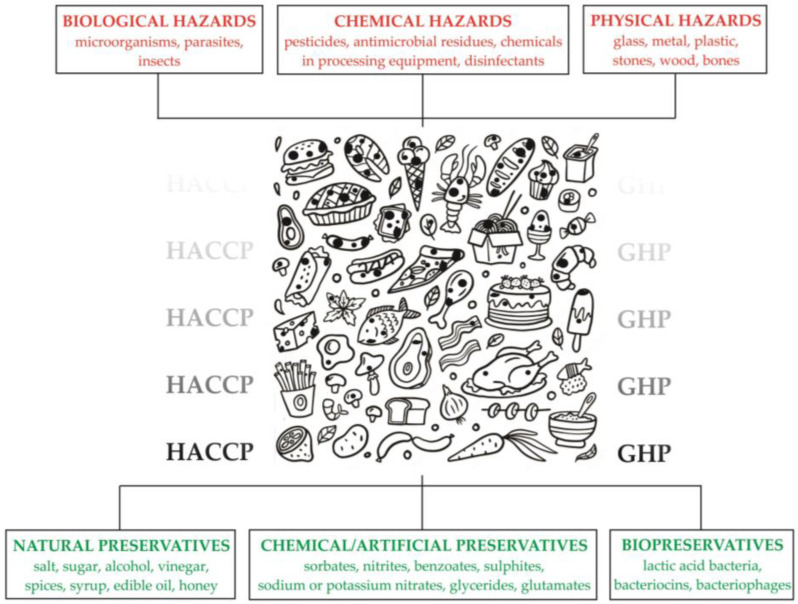
A brief overview of food safety hazards and measures to overcome them.

Food contamination refers to the condition in which food is no longer suitable for consumption due to the presence of undesirable biological, chemical, or physical agents (Figure 1). Biological hazards include microorganisms (bacteria, viruses, yeasts, molds), parasites, and insects [1]. Some of them are pathogenic or can produce toxins. Pathogenic microorganisms cause diseases that can vary in severity, extent, and consequences. Chemical hazards can come from pesticides or antimicrobial residues, chemicals in processing equipment, or disinfectants. Physical hazards include hard or sharp objects such as glass, metal, plastic, stones, wood, and bones, which can cause choking, cuts, or broken teeth. There are also undesirable foreign objects (hair, insects, and sand), but these are less likely to cause injury. Finally, a fourth type of contaminant is allergens (e.g., gluten) [2]. Food contamination can be primary (by air, soil, polluted waterways, pesticides used in agriculture, mycotoxins) or secondary (during production, transportation, storage, originating from packaging—primary packaging material or ambient conditions—polluted environment). It is therefore important to know all elements and carry out a risk assessment, as well as necessary to implement and comply with the Hazard Analysis Critical Control Point (HACCP) system and Good Hygiene Practices (GHP) during the preparation, storage, distribution, and serving of food [3].

Food is a suitable medium for the growth and proliferation of microorganisms that affect its appearance, taste, smell, and other characteristics by causing putrefaction, fermentation, rancidity, the production of toxins and pigments, and the appearance of mucus [4]. Worldwide, significant progress has been made in improving food preservation and safety techniques. Nevertheless, outbreaks of disease associated with foodborne pathogens (primarily bacteria, fungi, and viruses) continue to occur, making these pathogens a significant public health threat [5]. One of the major challenges in the food industry is the formation of microbial biofilms on various surfaces, utensils, equipment, and devices used in food production, which are difficult to remove. Compared to free-living planktonic, i.e., single bacterial cells, bacteria in biofilms are 100 to 1000 times more resistant [6].

The prevention of microbial contamination is essential to reducing the incidence of foodborne diseases. Contamination can be controlled by proper cleaning and sanitation. Equipment should be carefully designed to avoid microbial growth on all parts. An effective method to prevent microbial contamination in the industry would be early detection of microbial growth, especially to prevent biofilm formation. Ozone is used in solid foods to decontaminate and remove microorganisms. Thermal and non-thermal techniques such as microwave heating, pulsed electric field technology, high-pressure processing, high-intensity light technology, ohmic heating, ultrasonic techniques, and pulsed X-rays have recently been used for the preservation of liquid food in the industry [7].

Due to the increasing resistance of microorganisms to chemical agents and the decreasing effectiveness of synthetic preservatives, there is a growing need for alternative sources of natural, bioactive compounds with antimicrobial activity. The incorporation of probiotics and plant extracts into food formulations not only enriches foods with microorganisms and phytochemicals with biologically active compounds but also provides a means of product preservation. To achieve this goal, this review provides a concise overview of the basic facts on microbial contamination and preservation of food and presents many examples of natural preservatives, primarily beneficial bacteria and their metabolites, collected by searching the *PubMed*, *Scopus*, and *Google Scholar* databases from the past three decades, with a focus on the last 5 years. By summarizing numerous studies, identifying research challenges and regulatory barriers to their wider use, and outlining future research directions, this article makes an original contribution to the field of bioconservation [8].

## 2. Most Common Microbial Food Contaminants

Over 250 toxins and pathogens—a number that continues to grow—are transmitted through food [9]. Foodborne diseases are usually infectious (alimentary infection) or toxic (alimentary intoxication/toxoinfection) and are caused by pathogens that enter the body through contaminated food [10]. According to the US Centers for Disease Control and Prevention (CDC), approximately 48 million people contract foodborne diseases each year, of which 128,000 are hospitalized and 3000 are fatal [11]. There are 31 known pathogens responsible for food poisoning. The problem, however, is that most cases of poisoning are attributed to “unspecified agents” where no specific pathogen has been identified.

Norovirus is the most common cause of foodborne disease, while *Salmonella*, the second most common pathogen, tops the list of hospitalizations and deaths. In the US alone, *Salmonella* claims 420 lives each year. *Salmonella* infections can lead to complications, including persistent diarrhea, which leads to fluid loss and dehydration. In some cases, bacterial translocation (leakage of bacteria from the gastrointestinal tract), sepsis, and death can occur. In contrast, with salmonellosis, there is only a 0.03% chance of death [11]. *Listeria monocytogenes* causes listeriosis and is characterized by a low number of patients (1600 cases per year in the US) but a high mortality rate (16%). Listeriosis can be non-invasive or invasive. The invasive form affects certain high-risk groups, including pregnant women, who have a 20-fold increased risk. Severe listeriosis can lead to complications such as septicemia, meningitis, miscarriage, stillbirth, and premature birth [11]. *Escherichia coli* strains are classified into six pathotypes associated with diarrhea, namely: Shiga toxin-producing *E. coli* (STEC) (also known as Verotoxin-producing *E. coli* (VTEC) or enterohemorrhagic *E. coli* (EHEC)) as the one most commonly associated with foodborne outbreaks, enterotoxigenic *E. coli* (ETEC), enteropathogenic *E. coli* (EPEC), enteroaggregative *E. coli* (EAEC), enteroinvasive *E. coli* (EIEC), and diffusely adherent *E. coli* (DAEC) [12]. Multiplex PCR is used for the identification and rapid diagnosis of enteric pathogens in patient feces samples [13]. In February 2024, a severe outbreak of *E. coli* O26 was reported in France, which was linked to cheese made from raw milk. At the end of 2023, 11 cases of hemolytic uremic syndrome (HUS) associated with kidney failure were reported in children. The corrective measure was the withdrawal of those batches of cheese from the market. Raw milk and cheese made from raw milk should not be consumed by young children, especially those under 5 years of age, pregnant women, and people with weakened immune systems.

Among fungi, the most common is *Aspergillus niger*, which is associated with the spoilage of white bread [14]. Spoilage of baked goods is mainly caused by molds, yeasts, and, less frequently, bacteria [15]. Mold growth can occur in the production environment and cause contamination after baking, leading to food spoilage [16,17]. Mold infestation can become a food safety issue as it leads to the production of mycotoxins. Consumer demand for organic and fresh food has prompted food manufacturers to eliminate or reduce the use of preservatives, salt, and sugar in food, increasing the microbiological safety risk [18]. Inadequate hygiene standards for surfaces used in food storage can favor the growth of microorganisms and lead to cross-contamination. In addition, the presence of airborne contaminants [19] or contaminated surfaces that come into contact with food [20] can affect the microbiological quality of bakery products.

## 3. Food Preservation Techniques

Traditional and modern preservation techniques are used. Traditional techniques include cooking, salting, drying, pickling, freezing, and fermentation, while modern techniques include pasteurization, vacuum packing, pulsed electric field technology, high-pressure freezing, ultrasound, ozonation, nanotechnologies to remove toxins, biopreservation, food additives, antioxidants, and natural antimicrobial additives such as nisin, reuterin, and pediocin [21,22,23,24].

## 4. Preservatives

### 4.1. Role and Types of Preservatives

Preservatives, a type of food additive, extend the shelf life of food by slowing or stopping the growth of microorganisms and the physical changes in color, texture, and flavor that lead to spoilage. They are divided into two groups: natural preservatives, or Class I, and chemical/artificial preservatives, or Class II. Class I includes salt, sugar, alcohol, vinegar, spices, syrup, edible oil, and honey, while Class II includes sorbates, nitrites, benzoates, sulfites, sodium or potassium nitrates, glycerides, glutamates, and others [25].

Further, they can be antimicrobial preservatives that inhibit the growth of bacteria or fungi, including mold, or antioxidants such as oxygen scavengers that prevent the oxidation of food ingredients. Common antimicrobial preservatives are calcium propionate, sodium nitrate, sodium nitrite, and sulfites (sulfur dioxide, sodium bisulfite, potassium hydrogen sulfite, etc.), while antioxidants are added to fatty and oily foods to prevent rancidity [26]. The preservatives approved by the European Food Safety Authority (EFSA) are listed in Table 1, updated on 23 January 2024 [27].

**Table 1:** Approved preservatives—E numbers, side effects, and use in food.

Type	E Numbers *	Preservatives	Side Effects	Food
Antimicrobials	E200	Sorbic acid	Urticaria and contact dermatitis, rarely [25]	Pickles, margarine, fruit juices, jams, cheese, baked goods, snacks [28]
	E202	Potassium sorbate		
	E210	Benzoic acid	Skin rash, asthma, and possible brain damage [29]	High-acid foods and fruit drinks, flavored fermented milk products, fruits and vegetables, confectionery, processed fish, and fishery products [28]
	E211	Sodium benzoate		
	E212	Potassium benzoate		
	E213	Calcium benzoate		
	E214	Ethyl p-hydroxybenzoate		Baked goods, beverages, dressings, relishes [28]
	E215	Sodium ethyl p-hydroxybenzoate		
	E218	Methyl p-hydroxybenzoate		
	E219	Sodium methyl p-hydroxybenzoate		
Antimicrobials and antioxidants	E220	Sulfur dioxide	Asthma, urticaria, angioedema, abdominal pain, diarrhea, anaphylaxis [30]	Dried fruits and vegetables, pickled vegetables, fruit juices, sausages, cider, vinegar, wine [30]
	E221	Sodium sulfite	Headaches, palpitations, allergies, asthma, cancer [25]	Dried fruits and fruits, molasses, fried or frozen potatoes, shrimp, and lobster [28]
	E222	Sodium hydrogen sulfite		
	E223	Sodium metabisulfite		
	E224	Potassium metabisulfite		
	E226	Calcium sulfite		
	E227	Calcium hydrogen sulfite		
	E228	Potassium hydrogen sulfite		
Antimicrobials	E234	Nisin	Non-toxic [31]	Pasteurized, flavored, and long-life milk, processed cheese, cheese, other dairy products, canned vegetables [31]
	E235	Natamycin	Skin rash, hives, itching, difficulty breathing, tightness in the chest, swelling of the mouth, face, lips, or tongue [32]	Dairy products, meats, cottage cheese, sour cream, yogurt, packaged salad mix [32]
	E242	Dimethyl dicarbonate	Carcinogenesis [33]	Alcoholic and non-alcoholic drinks [34]
	E243	Ethyl lauroyl arginate	Non-toxic at a daily intake of 0.5 mg/kg [35]	Heat-treated meat products [35]
Antimicrobials and antioxidants	E249	Potassium nitrite	Carcinogenic effects [36]	Meat products [28]
	E250	Sodium nitrite		
	E251	Sodium nitrate		
	E252	Potassium nitrate		
Antimicrobials	E280	Propionic acid	Non-toxic in the amounts permitted by EU regulation [37]	Bakery products, cheese, fruits [28]
	E281	Sodium propionate		
	E282	Calcium propionate		
	E283	Potassium propionate		
	E284	Boric acid	Diarrhea and internal organ damage [37]	Sturgeon eggs (i.e., caviar) [37]
	E285	Sodium tetraborate; borax		
	E1105	Lysozyme	Only in people with allergies to egg whites [37]	Cheeses [37]

* Codes used within the European Union (EU) to identify food additives.

An ideal preservative should have the following properties: (1) being non-irritating, (2) maintaining product consistency, (3) maintaining taste and health, (4) being non-toxic, (5) being physically and chemically stable, (6) being compatible with all other ingredients, (7) being a good antimicrobial agent, (8) being effective, and (9) having a longer shelf life [26]. Preservative manufacturers submit an application explaining the use of food additives and provide scientific evidence that they are safe for human consumption. The Food and Drug Administration (FDA) reviews the evidence and grants approval if the use of the additive is “reasonably certain not to cause harm” to the consumer. Generally Recognized as Safe (GRAS) additives such as salt, sugar, spices, vitamins, and monosodium glutamate (MSG) are considered safe by experts based on their long-standing use in food and published scientific evidence.

### 4.2. Side Effects of Preservatives

Long-term and excessive consumption of chemical preservatives is highly associated with (1) respiratory diseases (asthma and bronchitis), (2) allergies, (3) hyperactivity in children, (4) disruption of hormones and impairment of growth and development (one of the causes of being overweight in many children), (5) weakening of heart tissue, (6) obesity (due to the content of fatty acids), (7) teratogenicity, and (8) cancer (due to the content of synthetic antioxidants butylated hydroxyanisole (BHA) and butylated hydroxytoluene (BHT)) [24,25,37]. Health problems can be avoided by using natural food additives derived from plants, animals, and microorganisms [38]. This has increased interest in biopreservatives, natural microbiota, and antimicrobials that extend the shelf life and safety of food without the use of chemical ingredients.

## 5. Probiotics and Postbiotics

According to the definition endorsed by the WHO and FAO in 2001 and 2002, and updated by the ISAPP in 2014, probiotics are defined as “live microorganisms that, when administered in adequate amounts, confer a health benefit to the host” [39]. Although probiotics are effective in combating foodborne pathogens, there are challenges in characterizing and elucidating the underlying molecular mechanisms of action and in developing potential delivery strategies that could maintain the viability and functionality of the probiotic in the target organ [40].

The ISAPP has defined a postbiotic as “a preparation of inanimate microorganisms and/or their components that confers a health benefit to the host”. Due to the inability to transfer the antimicrobial resistance genes, postbiotics have an advantage over probiotics [41,42]. Postbiotics are useful molecules produced by probiotic bacteria and include microbial components (lipoteichoic acid, teichoic acid, cell wall peptidoglycan, and polysaccharides) and cellular metabolites (organic acids, bacteriocins, short-chain fatty acids (SCFAs), enzymes, and vitamins) [43]. The antibacterial mechanisms of postbiotics and their impact on the absorption of healthy substances, cancer prevention, mental health, and other potential therapeutic effects are considered [44].

Probiotics containing bacterial strains from the genera *Lactobacillus* (reclassified as of March 2020 into 25 genera, including 23 new genera and with 261 different species [45]) and *Bifidobacterium,* as well as the yeast *Saccharomyces cerevisiae* var. *boulardii,* are the most common on the market. Also worth mentioning is the research and development of next-generation probiotics (NGPs), so-called live biotherapeutic products (LBPs), which are intended not only for conventional use as food or dietary supplements but also for pharmaceutical use as advanced therapy for various chronic diseases and cancer. To date, several potential NGPs have been identified that exhibit health benefits, such as *Prevotella copri*, *Christensenella minuta*, *Parabacteroides goldsteinii*, *Akkermansia muciniphila*, *Bacteroides thetaiotaomicron*, *Faecalibacterium prausnitzii*, and *B. fragilis* [46]. Although NGPs have multiple advantages over conventional probiotics [47], their safety and efficacy in the human population have not been proven [48]. The addition of probiotic cultures to food contributes primarily to food enrichment and obtaining a functional product, but also to food preservation, which will be discussed in more detail.

### 5.1. Probiotics as Potential Biopreservatives

Biopreservation is a modern technique for preserving food and extending its shelf life by using natural or controlled microorganisms such as lactic acid bacteria (LAB) and bacteriophages, which inhibit food spoilage [49]. The biocontrol mechanism of LAB is based on the competition of probiotics with pathogens for nutrients and/or the inhibitory/bactericidal effect of various metabolites, in particular low-molecular antibacterial peptides—bacteriocins, also known as natural antibiotics. Bacteriophages are viruses that block the growth of specific bacteria by attacking the host DNA and/or exert an antimicrobial effect by lysing the host bacteria.

Probiotics as biological preservatives are a potential intervention strategy for the prevention and control of foodborne infections and biofilm formation in the food industry [50,51]. On the one hand, they are naturally present in many foods, which can allow long-term storage at temperatures above refrigerator temperature, and on the other hand, they are novel in some foods, i.e., added live microorganisms that produce antimicrobial substances—metabolites (bacteriocins, organic acids, dipicolinic acid, fatty acids, hydrogen peroxide, carbon dioxide, and aromatic compounds)—that inhibit growth or kill pathogenic microorganisms. The mechanisms of action of probiotic strains in the gastrointestinal tract are well studied, in contrast to their interaction with foodborne pathogens [52,53]. It is known that they are strain-specific and act only on certain types of pathogens, as well as that the presence of one or more probiotic strains in a fermented product may enhance the beneficial properties of the probiotic strains involved. Nevertheless, determining the appropriate dose, type, and combination of probiotics in the fight against pathogens still deserves special attention.

### 5.2. Screening of LAB Isolates—Potential Probiotics

Once identified, all isolates must be tested for safety to be further investigated as candidates—potential probiotic strains. After that, the strain-specific effect of each of them is proven (in vitro, in preclinical research, and in clinical trials). First, the sensitivity to antibiotics is tested to exclude resistant strains from further research, and then the virulence factor of the isolated bacteria is determined or excluded.

#### 5.2.1. Antibiotic Susceptibility Testing

The selection of antibiotics is based on EFSA recommendations, which ensure that the concentrations cover the defined limits for the selected LAB [54]. According to the criteria defined by the EFSA, the isolates can be classified as susceptible, moderately susceptible, or resistant. The minimum inhibitory concentration (MIC, μg/mL) of the antibiotics ampicillin, chloramphenicol, clindamycin, erythromycin, gentamicin, kanamycin, streptomycin, and tetracycline was determined using the broth microdilution method according to the Clinical and Laboratory Standards Institute [55]. To make the probiotic strain safe, i.e., to exclude the possibility of horizontal and vertical transfer of resistance genes, especially to pathogenic bacteria, it must be sensitive to all antibiotics tested (phenotypic testing) or, if genotypic testing is performed, resistance genes must be absent, especially on plasmids.

#### 5.2.2. Virulence Factor Testing

Four groups of tests are included:

- Determination of biogenic amine-forming capacity [56];
- Production of hydrolytic enzymes: gelatinase, lipase, and DNase [57];
- Hemolytic activity [58];
- Presence of virulence genes encoding for the various virulence factors and amino acid decarboxylating enzymes: ace (collagen adhesion), hyl (hyaluronidase gene), asa1 (aggregation substance precursor), agg (aggregation substance), esp (enterococcal surface protein), gelE (gelatinase), efaAfs and efaAfm (cell wall adhesins), cylA, cylB cylM, cylLL, and cylLS (cytolytic activity), and hdc1, tdc, and odc (histidine, tyrosine, and ornithine decarboxylase activity, respectively) [58].

#### 5.2.3. Antimicrobial Activity Testing

One of the basic requirements for potential biological preservatives is the antimicrobial activity of the probiotic strains (probiotics) or their metabolites (postbiotics). In one study, 491 LAB isolates were tested for their antimicrobial activity against foodborne pathogens. Among them, six strains showed antimicrobial activity through potential bacteriocin production against 14 strains of *L. monocytogenes*, *Enterococcus faecalis* ATCC 29212, *Clostridium sporogenes* ESB050, and *C. perfringens* ESB054. Whole genome sequencing (WGS) identified the strains *Lactiplantibacillus plantarum* (previously known as *Lactobacillus plantarum* [45]) (2), *Leuconostoc mesenteroides* (1), and *Pediococcus acidilactici* (3), which produce bacteriocins. No virulence or antibiotic resistance genes were detected in the WGS analysis. None of these strains showed production of biogenic amines, gelatinase or DNAse, or hemolytic activity. Only *Lb. plantarum* 9A3 was sensitive to all tested antibiotics and showed bacteriostatic activity against four strains of *L. monocytogenes*. Therefore, this strain was selected for further investigation as it appears to be a strong candidate for potential application as a protective strain for the food industry. It is not only safe but also produces stable bacteriocins that inhibit important pathogens such as *L. monocytogenes* and *Clostridim perfringens* [58].

### 5.3. Probiotic-Pathogen Interaction

The interaction between probiotics and pathogenic microorganisms has been studied in vitro and in vivo (in animal models). One of the most important interactions is the competition for the cell binding site and the inhibition of pathogen growth, which prevents/reduces the colonization with the pathogen. For example, the probiotic strain *Streptococcus salivarius* K12, which produces salivabactin, showed an inhibitory effect on the human pathogen *S. pyogenes* in vitro and in vivo [59]. In rats infected with *L. monocytogenes*, suppression of the colonization of this pathogen was observed in the group treated with *Lb. casei* Shirota [60].

The main fermentation products that serve as preservatives are hydrogen peroxide, organic acids (lactic acid as the main metabolite, acetic acid, propionic acid, and phenyllactic acid), fatty acids (linoleic acid, butyric acid, caproic acid, and caprylic acid), and aromatic compounds (diacetyl, acetaldehyde, and reuterin). In addition to metabolites with bactericidal activity, some bacteria also produce metabolites with antifungal activity (cyclic dipeptides and phenylacetic acid). These metabolites are thermostable, with an optimal pH activity in the range of 3 to 4.5 [61]. Acetic acid has an inhibitory effect against some strains of *L. monocytogenes*, and a synergistic effect of acetic acid with lactic acid is known [62]. The antimicrobial effect of organic acids is due to the low pH of the substrate and the undissociated form of the acid molecule, which depends on the type of medium [63]. The mechanism of action of organic acids is based on the acidification of the cytoplasm by the passive diffusion of the undissociated, lipophilic acid through the cell membrane [64]. This leads to an interruption of the electrochemical proton gradient, resulting in an intracellular accumulation of anions, which leads to growth arrest or the death of the cell. Many LAB strains produce bacteriocins, i.e., antibacterial proteins, that are effective against foodborne pathogens such as *Staphylococcus aureus*, *Pseudomonas fluorescens*, *P. aeruginosa*, *S. typhi*, *Shigella flexneri*, *L. monocytogenes*, *E. coli* O157:H7, and *C. botulinum*. The bacteriocins secreted by LAB are thermostable and sensitive to proteases. They form pores in the cell membrane of the bacteria, which leads to increased permeability, inhibition of cell wall biosynthesis, and interruption of metabolic pathways [65,66].

Regarding biopreservation, it is of particular importance to further investigate the mechanisms of interaction of probiotic strains with foodborne pathogens. The antimicrobial effect of the metabolites of probiotic strains such as organic acids, bacteriocins, and hydrogen peroxide in food matrices is well known, but there are still challenges regarding the molecular mechanism of their action. In addition, it is necessary to determine the appropriate dose, type, and combination of probiotics (consortium) for the control of pathogens [52]. If several probiotic strains are used together (consortium), regardless of whether they are “multi-strains” (several strains within the same bacterial species) or “mixed species” (several strains belonging to different bacterial and yeast species), it must be tested during product development whether there is cross-inhibition between them. This is supported by numerous reports of randomly taken commercial probiotics of low quality, where, among other things, not all strains declared on the product can be identified [42,67,68]. If this is the case, such a consortium would not be recommended, as the probiotic strains would inhibit each other and their synergistic effect as a preservative, dietary supplement, or drug (pharmabiotic) would be absent.

Despite the preventive measures taken, foodborne diseases are still a global problem, and great efforts are being made to overcome them. The increasing resistance of many pathogens to antibiotics makes the discovery of alternative treatments or adjuvant therapies urgently necessary. Probiotics and their metabolites (postbiotics) have been recognized as a promising approach, and work is ongoing to find the best strains or combinations of strains of microorganisms (primarily bacteria and yeasts) that are effective in combating a particular pathogen [69]. In this context, here is a brief overview of the current state of knowledge on this topic for some of the most common foodborne pathogens, which is also summarized in Table 2.

#### 5.3.1. *Salmonella* spp.

Acute non-typhoidal salmonellosis (NTS), caused by *S. enterica* Typhimurium (STM), is one of the most common foodborne diseases. Previous studies have shown that the probiotic *Limosilactobacillus* (*Lactobacillus*) *reuteri* KUB-AC5 (AC5) exhibits anti-*Salmonella* activity in chickens by modulating the gut microbiota and immune response. However, the immunobiotic effect of AC5 on the mammalian host is still unknown. In a study by Buddhasiri et al. [70], the anti-*Salmonella* and anti-inflammatory effects of AC5 applied for 4, 7, and 11 days on STM infections were investigated using a mouse colitis model. Reduced proliferation and invasion of STM in the gut, together with attenuated intestinal inflammation and systemic dissemination, were observed in mice, especially after prolonged AC5 feeding and/or the combinatorial (direct and indirect inhibitory) effects of AC5 on STM.

The probiotic strains *Lb. plantarum* K132, *Lb. paracasei* K114, and *Lactococcus lactis* E124 showed remarkable in vitro anti-*Salmonella* activity in co-culture against *Salmonella* Typhimurium DT104, ranging from 96.5% growth inhibition (single culture of each probiotic strain) to 100% growth inhibition (mixed cultures of all three probiotic strains) [71]. In addition, the survival rate was significantly higher and the number of *Salmonella* in feces was significantly lower in mice treated with a mixture of these probiotic strains for 7 days. In another study, *Lb. casei* 5s isolated from Serbian homemade cheese was tested against *S. enterica* subsp. *enterica* serotype Abony [72]. It was shown that both the complete culture and the cell-free supernatants (CFSs) of *Lb. casei* 5s were able to inhibit the growth of *S. abony* NTCC 6017.

#### 5.3.2. *Escherichia coli*

*E. coli* is an important component of the human intestinal microbiota, but there are pathogenic strains (especially *E. coli* O157:H7) that cause various serious infections not only in the intestine. The most effective probiotics tested in vitro against *E. coli* were *B. animalis* subsp. *lactis* BB-12 and *Lb. reuteri* DSM 17938 as a single-strain probiotic and a mixture of lactobacilli, bifidobacteria, and enterococci as a multi-strain probiotic [73]. Although single-strain probiotics show antagonistic activity against *E. coli*, consortium probiotics have the advantage of being stronger, more resilient, and more effective. Oral isolates of *Lb. plantarum* G1 and *Lb. casei* G3 showed an antagonistic effect against *E. coli* ATCC 8739 [74]. In addition to the viable cells, the CFSs of *Lb. plantarum* G1 showed strong antimicrobial activity against *E. coli* and other tested strains. The indigenous isolate of *Lb. plantarum* G2 was also able to strongly inhibit the growth of *E. coli* ATCC 8739 [75].

One of the studies tested the antimicrobial activity of the probiotic strains *Lb. acidophilus* La-5 and *B. longum* ATCC 15707 and their metabolites (CFSs) against *E. coli* O157:H7 and *S. aureus* in yogurt and found that both probiotic strains showed an inhibitory effect on the growth of pathogens during fermentation and storage [76]. In vitro testing of *Lb. plantarum*, *Lb. gasseri*, *E. faecium*, *Bacillus subtilis*, and *Weissella paramesenteroides* strains using two methods (disk diffusion and well diffusion) showed inhibition of *E. coli* O157:H7, in contrast to the *E. coli* EHEC pathotype, where no antimicrobial activity of any of the tested strains was detected [77].

#### 5.3.3. *Yersinia* spp.

*Levilactobacillus brevis* 23017 is a selected probiotic strain that can regulate the immunity of the host animal and resist infections with pathogens. In mice infected with *Y. enterocolitica*, *Lb. brevis* 23017 prevented villi damage in the small intestine and slowed weight loss. Its protective role is to maintain a normal mucosal barrier by altering the expression of tight junction proteins and to stimulate the secretion of intestine-specific secretory immunoglobulin A by B cells via the regulation of cytokine and oxidative damage levels [78].

Bacteria isolated from the gut of healthy adult rainbow trout were tested for their probiotic properties and their inhibitory effect against *Y. ruckeri*. A total of 21 out of 541 isolates showed a zone of inhibition around at least one of the tested *Y. ruckeri* strains. The six were selected based on their ability to inhibit all pathogenic strains on solid media and were identified as *B. amyloliquefaciens* 131 and *Paenibacillus* spp. (codes 134, 1cc, 1d, 1k, and 2cc) [79]. In a similar study, bacterial isolates from rainbow trout and Nile tilapia were tested in vitro against *Y. ruckeri* and *Aeromonas salmonicida* subsp. salmonicida. Of the 369 isolates, 69 were selected after initial evaluation and 12 after an additional screening test (4 *P. acidilactici*, 7 *W. cibaria*, and 1 *W. paramesenteroides*), whereby only two isolates identified as *W. cibaria* were able to reduce the growth of pathogens [80].

#### 5.3.4. *Campylobacter* spp.

*C. jejuni* is one of the most common bacterial causes of gastroenterocolitis in humans worldwide, associated with the consumption of contaminated poultry, leading to diarrhea and other serious post-infectious complications. In the study by Dec et al. [81] on the probiotic potential of 46 *Lactobacillus* isolates from chicken feces or cloaca against *C. jejuni* and *C. coli*, *Lb. salivarius* and *Lb. reuteri* showed the highest anti-*Campylobacter* activity, with the reduced pH of the supernatant from the *Lactobacillus* culture playing a key role in inhibiting pathogen growth. Messaoudi et al. [82] described isolates of *Lb. salivarius* that were able to produce bacteriocins and also exhibited high anti-*Campylobacter* activity. The probiotic properties of five different *Lactobacillus* strains (*Lb. salivarius*, *Lb. johnsonii*, *Lb. reuteri*, *Lb. crispatus*, and *Lb. gasseri*) against *C. jejuni* were investigated in vitro by Taha-Abdelaziz et al. [83]. The difference in efficacy of the tested strains and the lack of a synergistic effect of the lactobacilli mixture were revealed.

Certain non-pathogenic strains of *B. subtilis* also show beneficial effects against *C. jejuni* in a chicken embryo as an in vivo model, which were strongly strain-dependent [84]. Other findings indicate that *B. subtilis* PS-216 reduces *C. jejuni* colonization and improves weight gain in poultry [85] and inhibits adhesion to abiotic surfaces and biofilm formation of *C. jejuni* [86], thus contributing to animal health and food safety.

#### 5.3.5. *Listeria monocytogenes*

*L. monocytogenes* is an important foodborne pathogen that poses a significant risk to public health and food safety. Although conventional physical and chemical methods are effective in inhibiting the growth of *L. monocytogenes* and prolonging the shelf life of food, the use of these methods usually leads to an undesirable deterioration in food quality. Recently, biologically based antimicrobial methods such as the use of probiotics have attracted much attention due to their promising antimicrobial effect and ability to maintain food quality [87].

The aim of the study, conducted from August 2021 to January 2022, was to identify the presence of *Listeria* spp. in various samples, including pasteurized milk, chicken filet, and stool samples from pregnant women admitted to outpatient clinics in Sharqia Governorate, Egypt. In addition, the study identified serotypes, virulence-associated genes, antibiotic resistance patterns, and biofilm formation in *L. monocytogenes* isolates, as well as the antibacterial and anti-biofilm activity of *Lb. plantarum* ATCC 14917 (*Lb. plantarum*) against *L. monocytogenes* isolates. In this study, virulent isolates of *L. monocytogenes* with a marked ability to form biofilms were identified in Egyptian foods, and treatment with CFS of *Lb. plantarum* was effective in reducing their numbers [88].

#### 5.3.6. Fungi

In one of the studies, a large number of LAB were isolated from traditionally fermented foods in India, and the biocontrol potential of the isolates was evaluated. A total of 20 LAB isolates were selected from the samples and tested for their antagonistic activity against *Fusarium verticillioides*. Among the 20 selected bioactive isolates, *Lacticaseibacillus brevis* MYSN105 and its CFS (corresponding to the postbiotic) showed the highest in vitro antifungal activity against *F. verticillioides*. In addition, *Lb. brevis* MYSN105 showed high tolerance to gastrointestinal conditions and adhesiveness to intestinal epithelial cells in vitro. The results suggest that *L. brevis* MYSN105 has promising probiotic properties and can potentially be used to develop biocontrol formulations to minimize contamination with *F. verticillioides* and improve food safety [89].

In another study described by Ali et al. [90], eight isolates: *Lb. plantarum*, *Lb. acidophilus*, *Lb. rhamnosus*, *Lb. salivarius*, *Lb. paracasei*, *B. longum*, *B. adolescentis*, and *B. breve* were tested for their antimicrobial activity, tolerance to low pH values, and sensitivity to antibiotics. In addition to testing the CFSs for antifungal activity using the indicator test strains of *A. niger*, *A. flavus*, *A. fumigatus*, *Penicillium chrysogenum*, and *Candida albicans*, the CFSs were also tested for antibacterial activity against *E. coli*, *S. aureus*, *Pseudomonas aeruginosa*, *E. coli* MC1400, and *L. ivanovii*. All isolates had an inhibitory effect, but in a different range, from mild to very strong.

**Table 2:** Summarized overview of probiotics and their postbiotics against foodborne pathogens.

Probiotic Strain	Foodborne Pathogen	Reference
*S. salivarius* K12	*S. pyogenes*	[59]
*Lb. casei* Shirota	*L. monocytogenes*	[60]
*Limosilactobacillus (Lactobacillus) reuteri* KUB-AC5 (AC5)	*Salmonella* spp.	[70]
*Lb. plantarum* K132 *Lb. paracasei* K114 *L. lactis* E124	*Salmonella* Typhimurium DT104	[71]
*Lb. casei* 5s isolate	*S. enterica* subsp. *enterica* serotype Abony	[72]
*B. animalis* subsp. *lactis* BB-12 *Lb. reuteri* DSM 17938	*E. coli*	[73]
*Lb. plantarum* G1 isolate *Lb. casei* G3 isolate	*E. coli* ATCC 8739	[74]
*Lb. plantarum G2* isolate	*E. coli* ATCC 8739	[75]
*Lb. acidophilus* La-5 *B. longum* ATCC 15707 and their metabolites (CFSs)	*E. coli* O157:H7 *S. aureus*	[76]
*Lb. plantarum*, *Lb. gasseri*, *E. faecium*, *B. subtilis*, *W. paramesenteroides*	*E. coli* O157:H7	[77]
*Levilactobacillus brevis* 23017	*Y. enterocolitica*	[78]
*B. amyloliquefaciens* 131 *Paenibacillus* spp.	*Y. ruckeri*	[79]
*W. cibaria*	*Y. ruckeri* *A. salmonicida* subsp. salmonicida	[80]
*Lb. salivarius* *Lb. reuteri*	*C. jejuni* *C. coli*	[81]
*Lb. salivarius*	*C. jejuni*	[82]
*Lb. salivarius*, *Lb. johnsonii*, *Lb. reuteri*, *Lb. crispatus*, *Lb. gasseri*	*C. jejuni*	[83]
*B. subtilis*	*C. jejuni*	[84]
*B. subtilis* PS-216	*C. jejuni*	[85]
*Lb. plantarum* ATCC 14917	*L. monocytogenes*	[88]
*Lb. brevis* MYSN105 and its CFS	*F. verticillioides*	[89]
*Lb. plantarum*, *Lb. acidophilus*, *Lb. rhamnosus*, *Lb. salivarius*, *Lb. paracasei*, *B. longum*, *B. adolescentis*, *B. breve*	*A. niger*, *A. flavus*, *A. fumigatus*, *P. chrysogenum*, *C. albicans*, *E. coli*, *S. aureus*, *P aeruginosa*, *E. coli* MC1400, *L. ivanovii*	[90]

## 6. Role of Probiotics and Postbiotics as Biopreservatives in the Food Industry

Biopreservation based on probiotics and postbiotics as functional ingredients naturally present in or added to food is an increasingly useful approach in the food industry. It represents a natural preservation technology that is as effective, if not more effective, than conventional chemical preservatives, but certainly much safer for health as it has little or no harmful effects [8]. The advantages of biopreservation lie in the targeted control of certain microorganisms that spoil food without affecting beneficial microbes. The lower energy requirement, which contributes to energy savings and a reduction in greenhouse gas emissions, makes this method indispensable for sustainable food production [91]. There is a growing interest in the use of antimicrobial active packaging, i.e., the incorporation of antimicrobial compounds (organic acids, bacteriocins, inorganic substances, enzymes, proteins, plant extracts, and essential oils) into contact packaging materials (primary packaging) to maintain or extend the quality and shelf life of food [92].

Recent research also addresses the potential application of postbiotics in biopreservation, food packaging, and biofilm control [93]. In this context, an improvement in food preservation has been demonstrated by postbiotic metabolites such as γ-aminobutyric acid (GABA) and bacteriocin-like inhibitory substances (BLIS) produced by *Lb. brevis* C23 co-cultures in plant-based medium [94]. The advantages of using postbiotics over probiotic bacteria, from which they are produced, are: (1) clear chemical structure, extended shelf life (even up to 5 years), and safe dosing parameters [95], (2) greater stability and safety as their viability is not required for mass production or consumption [96], (3) greater resistance [97], (4) low-risk profile as they do not require the ingestion of billions of viable bacteria [98], (5) independence of their functionality from cell viability [99], (6) non-production with the strain in situ but incorporation into meals [100], (7) stability at different temperatures and pH ranges [101], and inability to transfer the antimicrobial resistance genes [41].

### 6.1. Mechanism of Antimicrobial Action of Functional Food Ingredients

The observed inhibitory effect of LAB against both Gram-positive and Gram-negative bacteria can be attributed to the release of antimicrobial components such as organic acids, diacetyl, hydrogen peroxide, reuterin, and bacteriocins [8]. Organic acids are produced by certain strains of the genus *Lactobacillus* (lactic acid, phenyllactic acid), *Acetobacter aceti* (acetic acid), *Propionibacterium* sp. (propionic acid), *Leuconostoc* (phenyllactic acid), and *Enterococcus* (phenyllactic acid) and exert effects through their undissociated molecules by inducing protein and enzyme denaturation and decreasing the cytoplasmic pH and membrane function [102,103]. Diacetyl is produced by several LAB species (*L. lactis* biovar. *diacetylactis*, *Lb. paracasei*, *Lb. bulgaricus*, and *S. thermophilus*), especially during the metabolism of citric acid, and acts by deactivating important enzymes by modifying the catalytic center [104]. Hydrogen peroxide is produced by some LAB (*L. lactis*, *Lb. bulgaricus*, *Lb. johnsonii*, and *Lb. acidophilus*) under anaerobic growth conditions, has a strong oxidizing capacity that dissolves cellular components, and exhibits antimicrobial activity against bacteria, molds, and viruses, including bacteriophages [105]. Reuterin is produced by *Lb. reuteri* and acts as an inactivator of essential enzymes such as ribonucleotide reductase [105]. Bacteriocins are complex proteins or peptides produced by various LAB species, including *L. lactis*, *S. thermophilus*, *Lb. acidophilus*, *Lb. plantarum*, *Lb. sake*, *Lb. curvatus*, *Lb. mesenteroides*, *Lb. carnosum*, *Lb. gelidum*, *P. acidilactici*, *P. pentosaceus*, *P. parvulus*, *E. faecalis*, *E. faecium*, and *B. bifidum,* and act as destabilizers of the cytoplasmic membrane, leading to the formation of pores, and inhibitors of cell wall, nucleic acid, and protein synthesis [106]. Gram-positive bacteria are generally more sensitive to bacteriocins, while Gram-negative bacteria are usually resistant [107]. It is evident that many LAB strains have more than one mechanism of antimicrobial action, which contributes significantly to their efficacy against pathogens. In addition to the antimicrobial components, the antimicrobial activity can be attributed to the production of exopolysaccharides, but this mechanism of action needs further investigation.

### 6.2. Overview of the Application of Functional Food Ingredients

Probiotic bacteria are found in a variety of functional foods, including milk, yogurts, cheeses, and mousses, but also in non-dairy products such as cereals, fruit and vegetable juices, chocolate, mayonnaise, and meat products. They not only contribute to the sensory quality of food but also serve as effective biopreservatives that extend the shelf life of food [8]. The positive effects of probiotics and postbiotics are primarily achieved by lactic and acetic acid and the resulting acidification of the food [108], as well as by the bacteriocins nisin and pediocin and the resulting disruption of the membranes of the target microflora [109]. When probiotics are added to food, postbiotics are formed as a result of their metabolic processes, and dead (inactive) probiotic cells and their decay products are also present. Several studies in the food industry have shown that probiotics and their metabolites can prevent the adhesion and subsequent formation of biofilms by pathogenic microorganisms. They can also disrupt already established biofilms formed by a variety of foodborne microorganisms, with *Lactiplantibacillus* and *Lacticaseibacillus* being the most commonly tested genera (Table 3), both in the form of probiotic cells and as sources of CFSs [51].

**Table 3:** Summarized overview of probiotics and their postbiotics in food.

Probiotic Strain	Pathogen	Food	Reference
*Lactiplantibacillus sakei*—postbiotic solution (organic acids, polysaccharides, and other minor metabolites)	*L. monocytogenes*	Beef filet	[110]
*L. lactis, Pediococcus—* viable cells	Spoilage bacteria, saprophytes	Refrigerated foods	[105]
LAB—viable cells, organic acids, bacteriocins	*C. botulinum*, *S. serovars*, *S. aureus*	Fresh meat, seafood, certain processed meat products	[111]
*Lactobacillus*, *Lactococcus*, *Leuconostoc*—viable cells	*Pseudomonas*	Fresh milk, meat, eggs, seafood	[104]
*Lb. plantarum* UTNCys5-4, *L. lactis* subsp. *lactis* Gt28— peptides	*E. coli*, *Salmonella*, *Shigella*	Pineapple	[112]
*L. mesenteroides* WK32— postbiotics	Coliform, aerobic mesophilic bacteria, molds	Ready-to-eat baby leafy vegetables	[113]
*Lb. plantarum* Cs, *Lb. acidophilus* ATCC 314—postbiotics	*S. aureus*, *A. niger*, *E. coli*, *A. flavus*	Home-processed tomato paste	[114]
*Lb. plantarum*—isolates	*Rhodotorula mucilaginosa*	Yogurt, orange juice	[115]
*Lb. plantarum*—viable cells, organic acids	Fungi	Dairy products	[116]
*Lactobacillus* sp. RM1—CFS	*A. parasiticus*	Wheat grains	[117]

*L. monocytogenes* is a challenging pathogen as it tolerates stressful conditions in food matrices (acidity, oxidative and osmotic stress, low or high temperatures, presence of bacteriocins and other preservatives), and one of the approaches to reducing the number of its viable cells in beef filet is the application of an aerosolized postbiotic solution of *Lactiplantibacillus sakei* [110]. Viable cells of mesophilic bacteria such as *L. lactis*, certain strains of *Lactobacillus*, and *Pediococcus* added to refrigerated foods (at temperatures below 5 °C) significantly suppress the growth of spoilage bacteria and saprophytes and reduce the growth of pathogenic bacteria even at 10–12 °C [105]. LAB has a positive effect on the control of pathogens such as *C. botulinum*, *S. serovars*, and *S. aureus* in fresh meat, seafood, and certain processed meat products [111]. Viable cells of the genera *Lactobacillus*, *Lactococcus*, and *Leuconostoc*, added to fresh milk, meat, eggs, and seafood during a refrigerated storage period of 4–10 days, inhibit the growth of psychrotrophic bacteria of the genus *Pseudomonas* by 90% or more [104]. Peptides produced by *Lb. plantarum* UTNCys5-4 and *L. lactis* subsp. *lactis* Gt28 inhibit the growth of pathogens in pineapple [112], while postbiotics from *L. mesenteroides* WK32 reduce the number of coliform, aerobic mesophilic bacteria, and molds in ready-to-eat baby leafy vegetables [113]. Home-processed tomato paste treated with postbiotics from *Lb. plantarum* Cs and *Lb. acidophilus* ATCC 314 had reduced the microbial load of *S. aureus*, *A. niger*, *E. coli*, and *A. flavus* and consequently extended the shelf life at room temperature by up to 25 days [114].

In addition to the antibacterial effect, studies by Crowley et al. [115] also pointed to the antifungal properties of *Lb. plantarum* isolates used as an additive to milk starters in yogurt and as an inoculant in orange juice, as well as their ability to inhibit the growth of the yeast *Rhodotorula mucilaginosa*. This is consistent with the findings of Erfani et al. [116], who conducted a systematic review of the relevant literature for the period from 2000 to 2022 and identified *Lb. plantarum* as one of the most effective probiotic bacteria with an antifungal effect against food spoilage fungi. CFS of a new *Lactobacillus* sp. RM1 also had antifungal properties against *A. parasiticus* in wheat grains [117].

### 6.3. Advantages and Disadvantages of Functional Food Ingredients

Probiotics and postbiotics in food can be considered functional ingredients with a dual function. On the one hand, they improve the nutritional value and longevity of food [118], and on the other hand, they have positive effects on human health. Some of the already confirmed health benefits are immunomodulation, i.e., inhibition (suppression of allergies and inflammation) or enhancement (strengthening the host’s defenses against infections) [119], anti-cancer, antioxidant, anti-inflammatory, and anti-obesity activity [120,121,122], lipid-lowering effect [72], and blood pressure reduction, especially postbiotic supplements of *Lactobacillus* and *Bifidobacterium* spp. [123]. In addition, exopolysaccharides as a postbiotic from the strain *Lb. plantarum* are proposed for introduction into functional foods and use as antitumor agents [124].

The presence of antibiotic resistance in various types of bacteria (beneficial and pathogenic) associated with fermented foods that are otherwise consumed mainly for their nutritional and health properties could have potentially far-reaching adverse effects on human health. Therefore, continuous monitoring and management strategies for the prevention and control of this resistance are of great importance [125]. Moreover, the application of postbiotics instead of probiotics should be a preferred approach to overcome this due to their inability to transfer the antimicrobial resistance genes [41].

Food can be contaminated with antibiotic-resistant bacteria and/or antibiotic resistance genes in various ways. One of these is the possible presence of resistance genes in bacteria intentionally added during food processing (starter cultures, probiotics, bioconserving microorganisms, and bacteriophages). Raw foods can be consumed without prior processing or preservation and therefore represent a significant risk for the transmission of antibiotic resistance to humans, as any resistant bacteria present are not killed. Food processing that kills bacteria reduces the risk of the transmission of antibiotic resistance [126]. Antibiotic resistance genes, particularly to tetracyclines, penicillins, chloramphenicol, clindamycin, kanamycin, ciprofloxacin, and macrolides such as erythromycin, have been found in LAB in various fermented foods, particularly in certain cheeses, fermented meats, and spontaneously fermented vegetables [127,128,129]. In addition, certain strains of *Lb. delbrueckii* subsp. *bulgaricus*, commonly used in yogurt cultures, have shown resistance to mycostatin, nalidixic acid, neomycin, polymyxin B, trimethoprim, colimycin, sulfamethoxazole, and sulfonamides [130]. The occurrence of multidrug resistance is not uncommon, and the presence of antibiotic resistance genes has been detected on plasmid and/or chromosomal DNA, indicating the possible role of LAB as a reservoir for the spread of antibiotic resistance on pathogenic bacteria in food and the environment [128].

Coagulase-negative *Staphylococcus* species, commonly found in fermented animal products such as certain meats, cheeses, and fermented fish products, have been associated with the presence of antibiotic resistance genes, usually to tetracyclines, penicillins, chloramphenicol, and macrolides [126,131], while resistance to ampicillin, trimethoprim-sulfamethoxazole, amoxicillin-clavulanic acid, and oxacillin was found in various Nigerian fermented foods [132]. In another study, *S. saprophyticus* isolates from fermented foods and clinical samples were found to have considerable resistance to lincomycin, erythromycin, and tetracycline [133]. Antibiotic resistance has also been detected in other pathogens: (1) *Enterococcus* spp., commonly found in Turkish white cheese, to streptomycin, erythromycin, oxacillin, and vancomycin [134], (2) *Salmonella* strains, found in meat and minced meat used for the production of fermented sausage, to amoxicillin, gentamycin, streptomycin, and tetracycline, (3) *L. monocytogenes* to amoxicillin, benzylpenicillin, tetracycline, and ciprofloxacin, and (4) *E. coli* strains to amoxicillin, neomycin, streptomycin, and tetracycline [125,126,135].

### 6.4. Regulatory Challenges and Barriers for the Application of Functional Food Ingredients

To ensure the safe consumption of fermented products, various international organizations have commented on the safe use of microbial cultures. In 2012, a joint Action Team of the International Dairy Federation (IDF) Standing Committees on Nutrition and Health (SCNH) and on Microbiological Hygiene (SCMH) revised the IDF 377-2002 Bulletin and proposed a rational for the evaluation of species that have been safely used in fermented foods in the past, which was published as Bulletin of the IDF 455-2012. This bulletin reported a list of microbial food cultures used in fermented foods based on currently available scientific evidence, including 82 bacterial species and 31 yeast and mold species. This included 195 bacterial species and 69 yeast and mold species from the filum *Archinobacteria* (genera *Bifidobacterium*, *Corynebacterium*, *Brachybacterium*, *Microbacterium*, *Arthrobacter*, *Kocuria*, *Micrococcus*, *Propionibacterium*, and *Streptomyces*), *Firmicutes* (genera *Bacillus*, *Carnobacterium*, *Enterococcus*, *Tetragenococcus*, *Lactobacillus*, *Pediococcus*, *Leuconostoc*, *Oenococcus*, *Weisella*, *Macrococcus*, *Staphylococcus*, *Lactococcus*, *Streptococcus*, *Acetobacter*, *Gluconacetobacter*, *Hafnia*, *Halomonas*, and *Zymomonas*). Within this long list, microorganisms from the filum *Firmicutes* and especially from the genera *Lactobacillus*, *Lactococcus,* and *Streptococcus* are the most commonly used. LAB, the most common microorganisms used for the fermentation of food and beverages, belong to these genera [136].

There are different probiotic categories (probiotics in food and probiotic dietary supplements) and their relevant regulations therefore depend on the intended use of the product (to maintain health or to prevent or cure a disease or its symptoms). Probiotics in foods such as probiotic yogurts and probiotic dietary supplements such as probiotic capsules do not always require a lengthy approval process before they can be placed on the market and are regulated by a regional/country-specific regulation. They are generally targeted at healthy populations to reduce the risk of disease or dietary management of a disease, and the claim of benefits is subject to different regulatory criteria depending on the jurisdiction [137].

It should be noted that the practical use of microbial biopreservatives in the food industry is regulated by various authorities worldwide, including the FDA and EFSA. The regulatory status of microbial biopreservatives may vary from country to country, and manufacturers must comply with the applicable regulations in each market where their products are sold. Striking the right balance between effective dose and potential toxicity is crucial when introducing these substances into food products.

## 7. Conclusions

Different probiotic strains show antimicrobial activity against a range of bacteria, yeasts, and fungi and have the potential to be used, individually or in a consortium, in biopreservation. The consumption of functional foods brings not only safety but also benefits for general health, especially for the gastrointestinal tract and the immune system. Therefore, future research should aim to find the most effective strain or combination of strains of microorganisms without cross-inhibition between them that should be added to foods to ensure their safety while complying with regulations. In addition, research should focus on the molecular mechanisms of action of each component/metabolite, both individually and in combination with others, especially in the use of postbiotics without probiotics.

## Data Availability

No new data were created or analyzed in this study. Data sharing is not applicable to this article.
